# QUCoughScope: An Intelligent Application to Detect COVID-19 Patients Using Cough and Breath Sounds

**DOI:** 10.3390/diagnostics12040920

**Published:** 2022-04-07

**Authors:** Tawsifur Rahman, Nabil Ibtehaz, Amith Khandakar, Md Sakib Abrar Hossain, Yosra Magdi Salih Mekki, Maymouna Ezeddin, Enamul Haque Bhuiyan, Mohamed Arselene Ayari, Anas Tahir, Yazan Qiblawey, Sakib Mahmud, Susu M. Zughaier, Tariq Abbas, Somaya Al-Maadeed, Muhammad E. H. Chowdhury

**Affiliations:** 1 Electrical Engineering Department, College of Engineering, Qatar University, Doha 2713, Qatar; tawsifur.rahman@qu.edu.qa (T.R.); 1017052037@grad.cse.buet.ac.bd (N.I.); amitk@qu.edu.qa (A.K.); abrar@bmpt.du.ac.bd (M.S.A.H.); maez33839@hbku.edu.qa (M.E.); a.tahir@qu.edu.qa (A.T.); yazan.qiblawey@qu.edu.qa (Y.Q.); sakib.mahmud@qu.edu.qa (S.M.); 2 College of Medicine, Qatar University, Doha 2713, Qatar; ym1707134@student.qu.edu.qa (Y.M.S.M.); szughaier@qu.edu.qa (S.M.Z.); 3 BioMedical Engineering and Imaging Institute (BMEII), Icahn School of Medicine at Mount Sinai, New York, NY 10029, USA; enamul.bhuiyan@mountsinai.org; 4 Department of Civil Engineering, College of Engineering, Qatar University, Doha 2713, Qatar; arslana@qu.edu.qa; 5 Urology Division, Surgery Department, Sidra Medicine, Doha 26999, Qatar; tabbas-c@sidra.org; 6 Department of Computer Science and Engineering, College of Engineering, Qatar University, Doha 2713, Qatar; s_alali@qu.edu.qa

**Keywords:** artificial intelligence, COVID-19, pre-screening, crowdsourcing application, deep learning, cough and breath sounds, spectrogram

## Abstract

Problem—Since the outbreak of the COVID-19 pandemic, mass testing has become essential to reduce the spread of the virus. Several recent studies suggest that a significant number of COVID-19 patients display no physical symptoms whatsoever. Therefore, it is unlikely that these patients will undergo COVID-19 testing, which increases their chances of unintentionally spreading the virus. Currently, the primary diagnostic tool to detect COVID-19 is a reverse-transcription polymerase chain reaction (RT-PCR) test from the respiratory specimens of the suspected patient, which is invasive and a resource-dependent technique. It is evident from recent researches that asymptomatic COVID-19 patients cough and breathe in a different way than healthy people. Aim—This paper aims to use a novel machine learning approach to detect COVID-19 (symptomatic and asymptomatic) patients from the convenience of their homes so that they do not overburden the healthcare system and also do not spread the virus unknowingly by continuously monitoring themselves. Method—A Cambridge University research group shared such a dataset of cough and breath sound samples from 582 healthy and 141 COVID-19 patients. Among the COVID-19 patients, 87 were asymptomatic while 54 were symptomatic (had a dry or wet cough). In addition to the available dataset, the proposed work deployed a real-time deep learning-based backend server with a web application to crowdsource cough and breath datasets and also screen for COVID-19 infection from the comfort of the user’s home. The collected dataset includes data from 245 healthy individuals and 78 asymptomatic and 18 symptomatic COVID-19 patients. Users can simply use the application from any web browser without installation and enter their symptoms, record audio clips of their cough and breath sounds, and upload the data anonymously. Two different pipelines for screening were developed based on the symptoms reported by the users: asymptomatic and symptomatic. An innovative and novel stacking CNN model was developed using three base learners from of eight state-of-the-art deep learning CNN algorithms. The stacking CNN model is based on a logistic regression classifier meta-learner that uses the spectrograms generated from the breath and cough sounds of symptomatic and asymptomatic patients as input using the combined (Cambridge and collected) dataset. Results—The stacking model outperformed the other eight CNN networks with the best classification performance for binary classification using cough sound spectrogram images. The accuracy, sensitivity, and specificity for symptomatic and asymptomatic patients were 96.5%, 96.42%, and 95.47% and 98.85%, 97.01%, and 99.6%, respectively. For breath sound spectrogram images, the metrics for binary classification of symptomatic and asymptomatic patients were 91.03%, 88.9%, and 91.5% and 80.01%, 72.04%, and 82.67%, respectively. Conclusion—The web-application QUCoughScope records coughing and breathing sounds, converts them to a spectrogram, and applies the best-performing machine learning model to classify the COVID-19 patients and healthy subjects. The result is then reported back to the test user in the application interface. Therefore, this novel system can be used by patients in their premises as a pre-screening method to aid COVID-19 diagnosis by prioritizing the patients for RT-PCR testing and thereby reducing the risk of spreading of the disease.

## 1. Introduction

The novel coronavirus-2019 (COVID-19) disease has infected 320 million and caused death to around 5.5 million people worldwide as of 15 January 2022 [1]. This has led to countries imposing strict lockdowns to reduce the infection rate, which has severely affected the economic and social lives of people. Mass vaccination has helped some countries, but some countries have entered into second and third waves of infection. Due to the emerging new variants, the pattern of infection and effectiveness of vaccination is still under question. The common symptoms of COVID-19 include fever, cough, shortness of breath, and pneumonia. People with a compromised immune system or elderly people are more likely to develop serious illnesses but the younger population is also affected, especially by the new variants [2,3,4,5,6]. 

Currently, diagnosis of COVID-19 is done by time-consuming, expensive, and expert-dependent reverse transcription-polymer chain reaction (RT-PCR) testing. This kit is not easily available in some regions due to a lack of adequate supplies, medical professionals, and healthcare facilities. Moreover, it requires patients to travel to a laboratory facility to be tested, thereby potentially infecting others along the way. Due to the delay in obtaining the results of RT-PCR, rapid antigen detection tests have also been used in many countries, but they suffer from low accuracy [7,8,9]. Recently, Artificial Intelligence (AI) has been implemented in the health sector widely [10], such as on chest X-rays [11,12,13,14] and computed tomography (CT) scans [15,16,17], which have also been used for early detection of COVID-19 and other lung abnormalities. Recently, electrocardiogram (ECG) trace images have been used with AI for the detection of COVID-19 and other cardiovascular diseases [18]. Hasoon et al. [19] proposed a method for classification and early detection of COVID-19 through image processing using X-ray images. The evaluation results showed high diagnosis accuracy, from 89.2% up to 98.66%. Alyasseri et al. [20] provided a comprehensive review of the deep learning and machine learning (ML) techniques for COVID-19 diagnosis from studies between December 2019 and April 2021. This paper included more than 200 studies that were carefully selected from several publishers, such as IEEE, Springer, and Elsevier. It provided COVID-19 public datasets established in and extracted from different countries. Al-Waisy et al. [21] proposed a novel hybrid multimodal deep learning system for identifying COVID-19 virus in chest X-ray (CX-R) images and termed it the COVID-DeepNet system. It aids expert radiologists in rapid and accurate image interpretation, and helps in correctly and accurately diagnosing patients with COVID-19 with an accuracy rate of 99.93%. Abdulkareem et al. [22] proposed a model based on ML and the Internet of Things (IoT) to diagnose patients with COVID-19 in smart hospitals. Compared with benchmark studies, the proposed SVM model obtained the most substantial diagnosis performance (up to 95%). Obaid et al. [23] proposed a prediction mechanism that uses a long short-term memory (LSTM) deep learning model that has been carried out on a coronavirus dataset that was obtained from the records of infections, deaths, and recovered cases across the world. Furthermore, they have stated that by producing a dataset which includes features (temperature and humidity) of geographic regions that have experienced severe virus outbreaks, risk factors, spatiotemporal analysis, and social behavior of people, a predictive model can be developed for areas where the virus is likely to spread. All of the above approaches would need the patient to go to a medical center to provide a sample or undergo testing [18]. However, the asymptomatic COVID-19 patients will not undergo any test until the disease reaches a level of concern. Therefore, these COVID-19 patients can easily spread the disease. Moreover, vaccinated patients, when infected by the virus, are often asymptomatic or show very mild symptoms, and can spread the disease very easily. Thus, there is a need for an early screening tool for such patients in the convenience of their homes. 

Machine learning has been used for many applications in the field of speech and audio [24,25,26], including machine learning techniques for spectrogram images [27,28,29]. It is used for the screening and early detection of different life-threatening diseases. It is stated that breathing, speech, sneezing, and coughing can be used by machine learning models to diagnose different respiratory illnesses such as COVID-19 [30,31,32]. Different body signals such as respiration or heart signals have been used by researchers to automatically detect different lung and heart diseases (such as wheeze detection in asthma [33,34,35]). The human voice has been used for early detection of several diseases such as Parkinson’s disease, coronary artery disease, traumatic brain injury, and brain disorders. Parkinson’s disease was linked to the softness of speech which can result from a lack of vocal muscle coordination [36,37]. Different voice parameters such as vocal frequency, vocal tone, pitch, rhythm, rate, and volume can be correlated with coronary artery disease [38]. Invisible illnesses such post-traumatic stress disorder [39], traumatic brain injury, and psychiatric conditions [40] can be linked with audio information. Human-generated audio can be used as a biomarker for the early detection of different diseases and can be a cheap solution for mass population screening and pre-screening. This becomes even more useful and comfortable to the user if it is related to their daily activities and the data acquisition can be done non-invasively.

Recent works have showed how respiratory sounds (e.g., coughing, breathing, and voice) from patients who tested positive for COVID-19 in hospitals differ from sounds of healthy people. Digital stethoscope data from lung auscultation is used as a diagnostic signal for COVID-19 [41], while the coughs 48 COVID-19 patients versus patients with other pathological coughs collected with phones were used to detect COVID-19 using an ensemble of CNN models [42]. In [11], speech recordings from hospitalized COVID-19 patients were used to automatically detect the health status of the patients. Thus, it is possible to identify whether a person is infected by the virus or not by utilizing respiratory signals like breath and cough sounds.

Data collection from COVID-19 patients is challenging due to the possibility of getting infected and the datasets are often not publicly available. McFarlane et al. [43] had stressed the need for a COVID-19 cough database which would help the development of an algorithm for detecting COVID from coughs. They used a database of 73 individual cough events from public media and named it NoCoCoDa. They stressed the need for uniformity/consistency in the dataset to help develop reliable algorithms. Grant et al. [44] have utilized crowd-sourced recorded speech, breath, and cough data from 150 COVID-19-positive cases to train a machine learning model. They investigated random forest and deep neural networks using mel-frequency cepstral coefficients (MFCCs) and relative spectral perceptual linear prediction (RASTA-PLP) features and have achieved a 0.7983 area under the curve (AUC) for detecting COVID-19 using speech sound analysis and a 0.7575 AUC for detecting COVID-19 using breathing sounds. Mouawad et al. [45] used MFCC features of cough and vowel ‘eh’ pronunciation from a dataset collected by the Corona Voice Detect project in partnership with Voca.ai and Carnegie Mellon University. They used XGBoost machine learning classifier and achieved an *F*1-score of 91% for cough and 89% for vowel “eh”. Erdogam and Narin [46] discussed the features of cough spectrogram data with the help of empirical mode decomposition (EMD), discrete wavelet transform (DWT) and the ReliefF algorithm on a dataset from a free-access site, achieving a 98.06% *F*1-score in detecting COVID from cough sounds. Pahar et al. in [47] have investigated machine learning classifiers, long short-term memory (LSTM), and convolutional neural network (CNNs), and found that the ResNet50 network of the Coswara dataset [48] and Sarcos dataset [49] achieved an AUC of 0.98. Imran et al. [42] proposed a mobile app called AI4COVID-19, which records 3 s of cough sounds to analyze automatically for the detection of COVID-19 within 2 min using transfer learning. The pipeline consists of two stages: cough detection and collection, and COVID-19 diagnosis. In the cough detection engine, a user must record 3 s of good quality cough sounds, and a mel spectrogram image of the wave is analyzed with a convolutional neural network (CNN). After the cough is detected, the system passes to the COVID-19 diagnosis to decide the result. It consists of three AI approaches, the deep transfer learning multi-class classifier (DTL-MC), the classical machine learning multi-class classifier (CML-MC), and the deep transfer learning binary-class classifier. Some key limitations of the current AI4Covide-19 are (1) limited training data, (2) limited data to generalize the model, (3) an AI model is not publicly available. In another study by Pal and Sankarasubbu [50], the authors investigated deep neural networks (DNNs) on a dataset in which 328 cough sounds had been recorded from 150 patients of four different types: COVID-19, asthma, bronchitis, and healthy. In the study, Pal and Sankarasubbu’s trained DNN could distinguish the COVID-19 coughs from others with an accuracy of 96.83% [50]. These studies confirm that COVID-19 coughs have a unique pattern. Bagad et al. [51] found that a pre-trained ResNet18 classifier could identify COVID-19 coughs with an AUC of 0.72 using COVID-19-confirmed cough samples collected over the phone from 3621 individuals. Laguarta et al. [52] had an AUC of 0.97 and a sensitivity of 98.5% with a pre-trained ResNet50 model for distinguishing COVID-19 coughs from non-COVID-19 patients using coughs which trained on 4256 subjects and tested on the remaining 1064 subjects [52]. Belkacem et al. [53] reported a complete hardware system that can be used to collect cough samples, temperature (via thermos camera) and airflow (via spirometer) and transmit this information to a database using smartphones. Next, cough samples and other health details with expert opinion were used to train a machine learning network to classify the samples as either COVID-19, bronchitis, flu, cold, or other. They used the existing motivation from recent papers that cough samples and machine learning networks are very useful in distinguishing between COVID-19 and healthy patients, but confirmed it with other data (airflow and body temperature). However, they have not mentioned the performance of their approach. A similar approach was adopted by Rahman et al. [54] utilizing chest X-rays, CT Scans, cough samples, temperature, and symptom inputs from patients. Although both the above approaches make the final results very reliable, they cannot be used immediately due to the hardware or extra health details needed for those systems.

Brown et al. [55] collected both cough and breathing sounds, then investigated how such data can aid with COVID-19 diagnosis. They provided handcrafted features for cough and breath sounds such as duration, onset, tempo, period, root mean square (RMS) energy, spectral centroid, roll-off frequency, zero-crossing, mel-frequency cepstrum (MFCC), and delta MFCC. Combined with deep transfer learning, VGGish, which is a convolution network designed to extract audio features, automatically achieved an accuracy of 0.80 ± 0.7 for two-class classification problems using the cough and breathing data. This dataset has also been used by Coppock et al. [56] in a pilot study, even before the dataset was made public, with their deep learning network achieving an AUC of 0.846. Kumar et al. [57], with their developed deep convolutional network, achieved a weighted *F*1-score of 96.46% in distinguishing between non-COVID and COVID-19 patients. This dataset was shared with our team under a data-sharing agreement, and was used to develop a machine learning pipeline in combination with Qatari data. 

The scope for having a more reliable and robust machine learning network trained and validated using a diverse database (due to limitations in terms of inconsistency and low-quality recordings in the available datasets) has motivated the current work. This work proposes a novel machine-learning framework using the combined Cambridge and Qatari cough and breathing sound databases. Most of the previous works either used classical machine learning with hand-crafted features or used pre-trained models to classify the spectrograms. A very limited number of works used combined datasets and no work has used the novel stacking concept for increasing model performance. Moreover, none of the AI-enabled data collection applications can show instant outcomes of the users’ data. Most applications are mere crowd data collection applications. We developed an AI-enabled web application as a pre-screening tool to decrease the pressure on health centers and provide a faster and more reliable testing mechanism to reduce the spread of the virus. Our contribution can be summarized as follows:

Conduct a literature review of related works to prove the potential applicability of the proposed solution. 

Point out the limitations of related works and how the proposed solution may overcome those problems. 

To the best of the authors’ knowledge, this is the first time an innovative and novel stacking-based CNN model using spectrograms of cough and breath sounds have been proposed.

Experimentally prove cough sounds have latent features to distinguish COVID-19 patients from non-COVID patients.

A web application with a backend server was created that allows the user to share symptoms and cough and breath data for COVID-19 diagnosis anonymously from a computer, tablet, or Android or iOS mobile phone. 

To the best of our knowledge, QUCoughScope (https://www.qu-mlg.com/projects/qu-cough-scope, accessed on 5 May 2021) is the first solution that is not just an application to collect crowd-sourced data. Rather, we have implemented a deep-learning pipeline in the backend to immediately provide the screening outcome to the user.

This article consists of six sections. In the introduction, we explained the problem of the current COVID-19 testing approach and how it can be addressed with the help of our pre-screening tool. Section II highlights related works, while Section III introduces the methodology, with details of the dataset, data preparation, and experiment, and Section IV summarizes all the results. Section V explains the implementation details while Section VI concludes the article. 

## 2. Methodology

**Figure 1:**
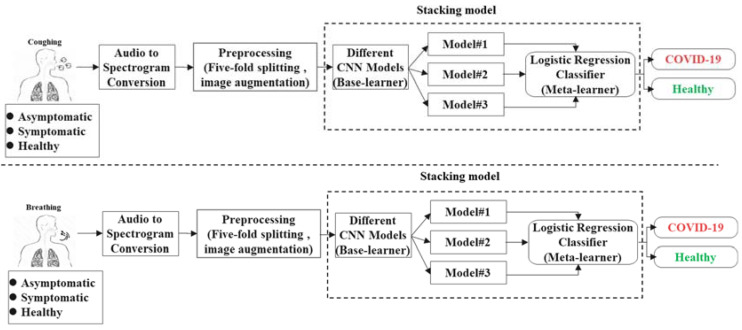
Methodology of the study.

The overall methodology of the study is summarized in Figure 1. This study used cough and breath sounds of COVID-19 (symptomatic and asymptomatic) and healthy subjects after converting these sounds into spectrograms to identify COVID-19 patients. This paper discusses four different binary classification experiments: healthy and COVID-19 symptomatic (i) and asymptomatic (ii) subjects using cough sound spectrograms; healthy and COVID-19 symptomatic (iii) and asymptomatic (iv) subjects using breath sound spectrograms. 

For all four experiments, novel stacking machine learning models were deployed, in which the eight CNN models were used as the base learners and then a logistic regression-based meta learner was used to detect COVID-19 from cough and breath sound spectrograms. Detailed descriptions of the dataset, preprocessing, and the experiments are presented below.

### 2.1. Dataset Description

Several public datasets are available such as Coswara [48], CoughVid [58], and the Cambridge dataset [55]. However, the Cambridge dataset was not completely public, and the team has made it available upon request. Among the accessible datasets, the Cambridge dataset was the most reliable as it was acquired in a well-designed framework. Moreover, the authors have collected a similar cough and breath dataset from COVID-19-infected and healthy subjects with our proposed framework. 

Cambridge dataset: The Cambridge dataset was designed for developing a diagnostic tool for COVID-19 based on cough and breath sounds [55]. The dataset was collected through an app (Android and web application (www.covid-19-sounds.org (accessed on 5 May 2021))) that asked volunteers for samples of their coughs and breathing as well as their medical history and symptoms. Age, gender, geographical location, current health status, and pre-existing medical conditions were also recorded. Audio recordings were sampled at 44.1 kHz and subjects were from different parts of the world. Cough and breath sound samples were collected from 582 healthy subjects and 141 COVID-19-positive patients. Among them, 264 healthy subjects and 54 COVID-19 patients had cough symptoms while 318 healthy subjects and 87 COVID-19 patients had no symptoms (Table 1). 

Qatari dataset: The QU cough dataset [59] consists of both cough and breath data from symptomatic and asymptomatic patients. Cough and breath sound samples were collected from 245 healthy subjects and 96 COVID-19-positive, respectively. Among them, 32 healthy subjects and 18 COVID-19 patients had cough symptoms while 213 healthy subjects and 78 COVID-19 patients had no symptoms (as shown in Table 1). 

In this study, we investigated the cough and breath sounds to overcome the limitations of some related works. We have therefore investigated two different pipelines for cough and breath. Moreover, for both cough and breath, we investigated symptomatic and asymptomatic patients’ data. Both datasets were merged to train, validate, and test the models in this study. Table 2 shows the experimental pipelines used in this study. 

### 2.2. Pre-Processing Stage

As shown in Figure 1, the input data (i.e., user cough and breath sounds) were converted to spectrograms, which were then tested using a 5-fold cross validation approach with 80% for training and 20% for testing. The detailed pre-processing stage is mentioned below:

#### 2.2.1. Audio to Spectrogram Conversion

**Figure 2:**
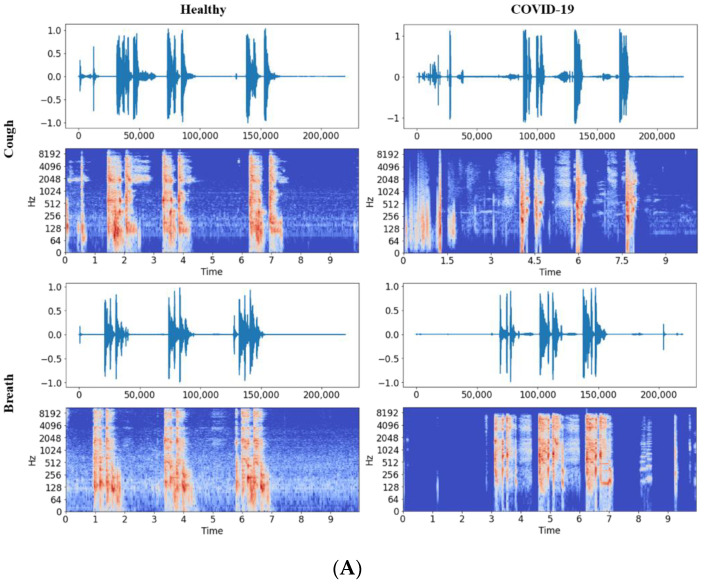

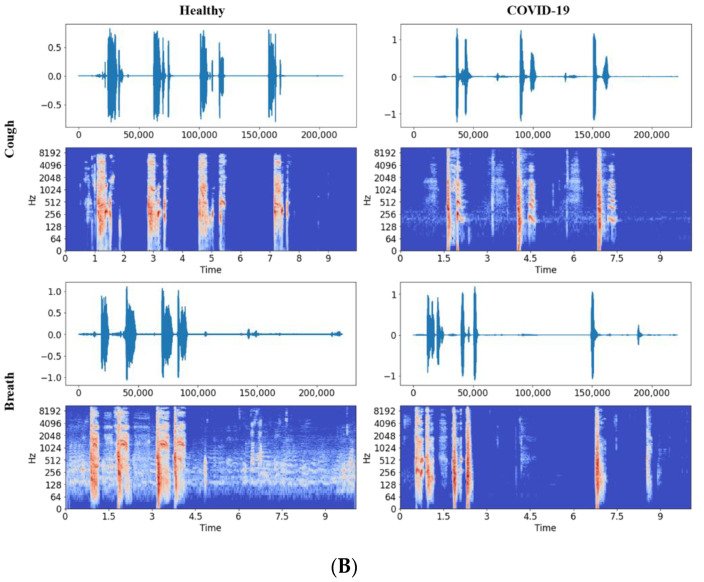
Cough and breath sound waveforms and spectrograms for (**A**) symptomatic and (**B**) asymptomatic healthy subjects and COVID-19 patients.

Since the dataset was collected using web and Android platforms, it was first organized into two sub-sets: cough and breath sounds. Then, each of these subsets was subdivided into symptomatic and asymptomatic groups. Each of the symptomatic and asymptomatic breath and cough sounds for COVID-19 and healthy groups were visualized in the time domain to see potential differences among them (Figure 2). 

Firstly, we converted cough and breath sounds to spectrograms. A spectrogram is a visual representation of an audio signal that shows the evolution of the frequency spectrum over time. A spectrogram is usually generated by performing a Fast Fourier Transform (FFT) on a collection of overlapping windows extracted from the original signal. The process of dividing the signal in short-term sequences of fixed size and applying FFT on those independently is called short-time Fourier transform (STFT). The spectrogram is the squared magnitude of the STFT of the signal, s(t) for a window width, w. These are the parameters used for STFT: n_fft = 2048, hop_length = 512, win_length = n_fft, and window = ‘hann’. 

#### 2.2.2. Five-Fold Cross-Validation 

The training dataset had to be balanced to avoid biased training. This was done with the help of the data augmentation approach, an effective method for providing reliable results evident in many of the authors’ recent publications [11,12,60,61,62,63]. In this study, two augmentation strategies (scaling and translation) were utilized to balance the training images shown in Table 3. The scaling operation is the magnification or reduction of the frame size of the image; 2.5% to 10% image magnifications were used in this work. Image translation was done by translating images horizontally and vertically by 5% to 10%. The complete image set was divided into 80% training and 20% testing sub-sets for five-fold cross-validation, and 10% of training data were used for validation, whose primary purpose was to avoid model overfitting. Table 3 shows the number of training, validation, and test images used in the two experiments on symptomatic and asymptomatic patients.

As discussed earlier, eight pre-trained CNN models were used in the study and were implemented using PyTorch library with Python 3.7 on an Intel^®^ Xeon^®^ CPU E5-2697 v4@ 2.30 GHz and 64 GB RAM, with a 16-GB NVIDIA GeForce GTX 1080 GPU. Eight of the pre-trained CNN models were trained using the same training parameters and stopping criteria mentioned in Table 4. Five-fold cross-validation results were averaged to produce the final receiver operating characteristic (ROC) curve, confusion matrix, and evaluation matrices. Here, 80% of the images were used for training and 20% for testing per fold. Image augmentations were used in the training set, and 20% of the non-augmented training set was used for validation to avoid overfitting of the models [64]. We also used a logistic regression classifier as a meta-learner for the final prediction in the stacking model where ‘lbfgs’ solver with L2 regularization was used and the maximum iteration was 100.

### 2.3. Stacking Model Development

**Figure 3:**
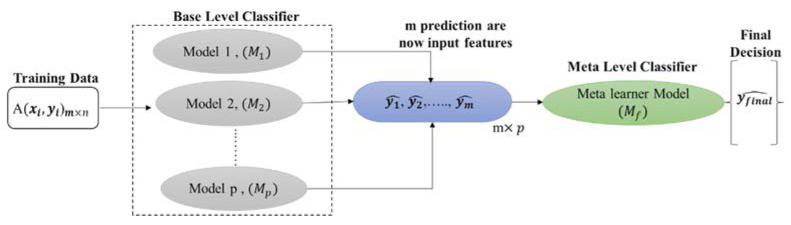
Stacking model architecture.

In this study, we used a CNN-based stacking approach in which the eight state-of-the-art CNN models (Resnet18 [65], Resnet50 [65], Resnet101 [65], InceptionV3 [65], DenseNet201 [66], Mobilenetv2 [67], EfficientNet_B0 [68], and EfficientNet_B7 [68]) were used as a base learner and multiple best-performing models were used to train a logistic regression based meta learner classifier for the final decision. A single dataset A consists of data vectors ($x_{i}$) and their classification score ($y_{i}$). At first, a set of base-level classifiers $M_{1},\ldots \ldots ,M_{p\;}$ is generated and the outputs are used to train the meta-level classifier, as illustrated in Figure 3.

We used five-fold cross-validation to generate a training set for the meta-level classifier. Among these folds, base-level classifiers were used on four folds, leaving one fold for testing. Each base-level classifier predicts a probability (0 to 1) over the possible class values. Thus, using input x, a probability distribution is created using the predictions of the base-level classifier set M:

(1) $${\;P}^{M}\left(x\right)=\left(P^{M}\left(c_{1}|x\right),P^{M}\left(c_{2}|x\right),\ldots \ldots .,P^{M}\left(c_{p}|x\right)\right)$$

where $\left(c_{1},c_{2},\ldots \ldots ,c_{p}\right)$ is the set of possible class values and $P^{M}\left(c_{i}|x\right)$ denotes the probability that example x belongs to a class $c_{j}\;$ as estimated (and predicted) by classifier M in Equation (1). The class $c_{i}$ with the highest-class probability is predicted by classifier M_j_. The meta-level classifier $M_{f}$ and attributes are thus the probabilities predicted for each possible class by each of the base-level classifiers, i.e., $P^{M_{j}}\left(c_{i}|x\right)$ for i = 1,…., p and j = 1,…., N. The pseudo-code for the stacking approach is shown in Algorithm 1.
**Algorithm 1:** Stacking classification**Input:**$\mathrm{training}\;\mathrm{data}\;D={\left\{x_{i},\;y_{i}\right\}}_{i=1}^{m}$ **Output:** a stacking classifier H 1: Step 1: learn base-level classifiers 2: **for** t = 1 to T **do** 3: $\;\;\;\;\mathrm{learn}\;h_{t}$ based on D 4: **end for** 5: Step 2: construct new data set of predictions 6: **for** i =1 to m **do** 7: $\;\;\;\;D_{h}=\left\{x_{i}{}^{\prime },{\;y}_{i}\right\}$$,\;\mathrm{where}\;x_{i}{}^{\prime }=\left\{h_{1}\left(x_{i}\right),\ldots \ldots ,h_{T}\left(x_{i}\right)\right\}$ 8: **end for** 9: Step 3: learn a meta-classifier 10: $\mathrm{learn}H\;\mathrm{based}\;\mathrm{on}\;D_{h}$ 11: **return** H

### 2.4. Performance Metrics

To evaluate the performance of the COVID-19 detection classifiers, we used the receiver operating characteristic (ROC) and area under the curve (AUC) along with precision, sensitivity, specificity, accuracy, and *F*1-Score as shown in Equations (2)–(6). Here, *TP*, *TN*, *FP*, and *FN* represent the true positive, true negative, false positive, and false negative, respectively.

(2) $$Accuracy=\frac{TP+TN}{TP+TN+FP+FN}$$

where accuracy is the ratio of the correctly classified samples to all the samples.

(3) $$Precision=\frac{TP}{TP+FP}$$

where precision is the rate of correctly classified positive class samples among all the samples classified as positive.

(4) $$Sensitivity=\frac{TP}{TP+FN}$$

where sensitivity is the rate of correctly predicted positive samples in the positive class samples,

(5) $$\;F1=2\frac{Precision\times Sensitivity}{Precision+Sensitivity}$$

where *F*1 is the harmonic average of precision and sensitivity.

(6) $$Specificity=\frac{TN}{TN+FP}$$

where specificity is the ratio of accurately predicted negative class samples to all negative class samples. 

The performance of deep CNNs was assessed using different evaluation metrics with 95% confidence intervals (CIs). Accordingly, the CI for each evaluation metric was computed, as shown in Equation (7):

*r*=*z*√*metric*(1 − *metric*)/N (7)

where *N* is the number of test samples, and *z* is the level of significance that is 1.96 for 95% CI.

In addition to the above metrics, the various classification networks were compared in terms of elapsed time per image, or the time it took each network to classify an input image, as shown in Equation (8).

∆T = T_2_ − T_1_ (8)

In this equation, T_1_ is the starting time for a network to classify a cough sound, S and T_2_ is the end time when the network has classified the same cough sound, S.

## 3. Results and Discussion

This section describes the performance of the different classification networks on healthy and COVID-19 cough and breath sound spectrograms for symptomatic and asymptomatic patients. As mentioned earlier, two different experiments using cough and breath sound spectrograms were conducted: (i) symptomatic COVID-19 and healthy, and (ii) asymptomatic COVID-19 and healthy. The comparative performance of different CNNs for these classification schemes is shown in Table 5A,B.

Overall accuracies for five-fold cross-validation from the top three CNN models for symptomatic and asymptomatic patients using cough sounds are 95.38%, 94.29%, and 93.25% and 98.5%, 98.28%, and 96.84%, respectively. The top three networks for symptomatic and asymptomatic patients using cough sounds are Resnet50, Resnet101, and DenseNet201 and Mobilenetv2, DenseNet201, and Resnet101, respectively. On the other hand, the overall accuracies from the top three CNN models for symptomatic and asymptomatic patients using breath sounds are 90.33%, 87.57%, and 84.53% and 75.6%, 69.72%, and 68.4%, respectively. The top three networks for symptomatic and asymptomatic patients using breath sounds are EfficientNet_B0, MobileNetv2, and ResNet101 and EfficientNet_B7, ResNet101, and MobileNetv2, respectively. It is evident from the results that cough sound-based stratification models perform better than breath sound-based models, for both symptomatic and asymptomatic patients.

Interestingly, the stacking CNN model outperformed all CNN models for both cough and breath sounds, as can be seen from Table 5. It achieved accuracies of 96.5% and 98.85% for symptomatic and asymptomatic patients’ cough sounds, respectively. On the contrary, it produced accuracies of 91.03% and 80.01% for symptomatic and asymptomatic patients’ breath data, respectively. It is clear that breath sounds were unable to classify healthy subjects and COVID-19 patients reliably, whereas cough sounds performed better for both symptomatic and asymptomatic patients. 

**Figure 4:**
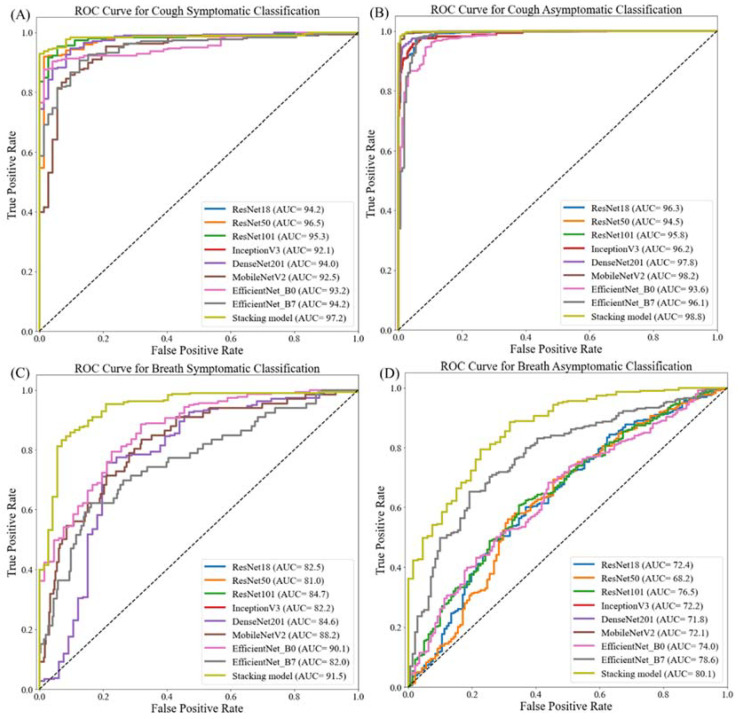
ROC curve for healthy and COVID-19 patients’ classification using cough sounds for (**A**) symptomatic patients and (**B**) asymptomatic patients, and using breath sounds for (**C**) symptomatic patients and (**D**) asymptomatic patients.

Figure 4 shows the area under the curve (AUC)/receiver-operating characteristic (ROC) curve (also known as AUROC (area under the receiver-operating characteristic)) for the symptomatic and asymptomatic patients’ cough and breath data. These ROC curves clearly depict that the stacking model performs better than any individual CNN model for cough and breath data, however, as mentioned earlier, cough sounds can reliably distinguish COVID-19 patients from the healthy group. It can also be seen that the best-performing scheme is the asymptomatic COVID-19 patients’ stratification using cough sounds. The asymptomatic patients are the ones who are spreading the virus unknowingly, and our trained network performs well in detecting them from their cough sounds. Therefore, this COVID-19 screening framework can significantly help in screening suspected populations and reducing the risk of spread. 

**Figure 5:**
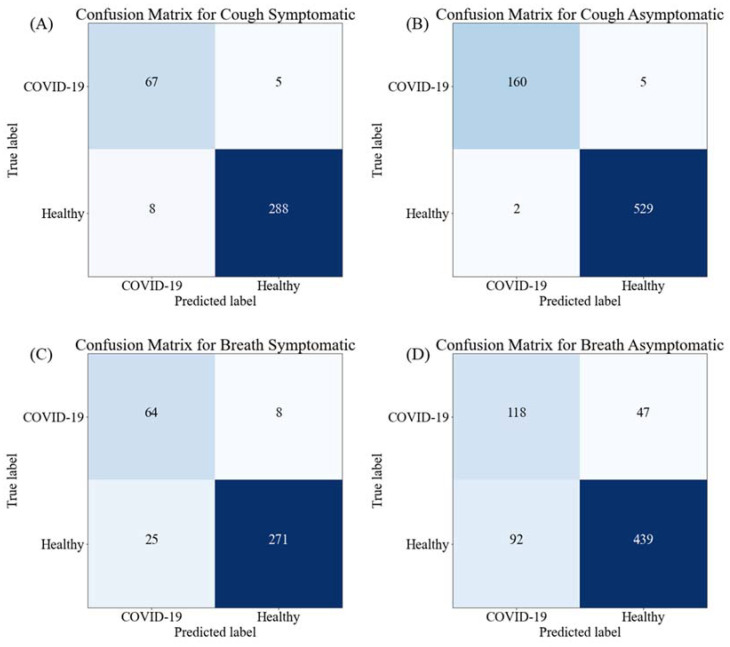
Confusion matrices for healthy and COVID-19 classification using cough sounds for (**A**) symptomatic patients and (**B**) asymptomatic patients, and using breath sounds for (**C**) symptomatic patients and (**D**) asymptomatic patients using best performing stacking CNN models.

Figure 5 shows the confusion matrix for the outperforming-stacking model for the cough data of symptomatic and asymptomatic patients and the breath data of symptomatic and asymptomatic patients. It can be noticed that even with the best-performing model, eight out of 72 COVID-19 spectrogram images were miss-classified as healthy and 9 out of 296 healthy spectrogram images were mis-classified as COVID-19 images for symptomatic cough sound spectrogram images. On the other hand, five out of 165 COVID-19 images were mis-classified as healthy and only two out of 531 healthy spectrogram images were mis-classified as COVID-19 images for asymptomatic cough sound spectrogram images. Once again, consistent with the results from Figure 4, the cough sounds performed very well in distinguishing between the asymptomatic subjects. 

For symptomatic breath sound spectrogram images, eight out of 72 COVID-19 images were miss-classified as healthy and 25 out of 296 healthy spectrogram images were mis-classified as COVID-19 images while 47 out of 165 COVID-19 images were mis-classified as healthy and 92 out of 531 healthy spectrogram images were mis-classified as COVID-19 images for asymptomatic breath sound spectrogram images. It is evident from the confusion matrices that the cough sound spectrogram outperformed the breath sound spectrogram. This outstanding performance of any computer-aided classifier using non-invasively acquirable cough sounds can significantly help with fast diagnosis of COVID-19 immediately and in the comfort of the user’s home.

**Figure 6:**
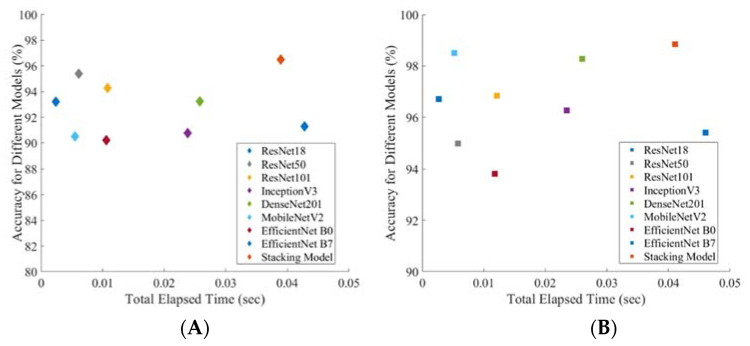
Accuracy vs inference time plot for binary classification using (**A**) symptomatic cough sound spectrograms, and (**B**) asymptomatic cough sound spectrograms.

Figure 6 shows a comparison of accuracy versus the inference time for each image for different CNN networks and the stacking CNN model for symptomatic and asymptomatic data. Inference times of the best-performing stacking network for symptomatic and asymptomatic cough sounds were about 0.0389 and 0.0411 s, respectively. Even though the inference time for the stacking model was higher than for most of the individual models, the inference time was still small enough to be suitable for real-time applications [69]. Therefore, to enable real-time application, we have deployed the best-performing stacking models in a web application that can be used from any mobile browser to make it independent from Android and iOS platforms. The next section describes the development and deployment steps of the AI-enabled web application. 

### AI-Enabled Application for COVID-19 Detection

An AI-enabled application was developed using Flutter, a cross-platform app development framework maintained by Google which uses the Dart programming language. The utility of using a cross-platform framework over native frameworks like Swift or Kotlin is that we can maintain multiple platforms like Android, iOS, and even desktop using a single codebase. This will in essence provide us with the maximum coverage for users, quicker development and continuous integration, seamless deployment and maintenance, easier cloud integration, and increased stability. Furthermore, using Flutter instead of other cross-platform frameworks like Ionic comes with the benefit of developing almost near-native code with complete access to native plugins and device hardware features in device AI using built-in GPU. We deployed an application entitled QUCoughScope [70] that allows patients to upload cough and breath sounds along with clinical history. For our purposes, the application requires access to the microphone of the smartphone and records cough and breath sounds. The mobile-recorded audio signal and symptoms, once received by the server machine, undergo an STFT operation to convert raw audio signals into spectrogram images without any pre-processing. The deployed Google computation engine-based backend AI-based server analyzes the uploaded sounds to classify them as healthy or COVID-19-positive. 

**Figure 7:**
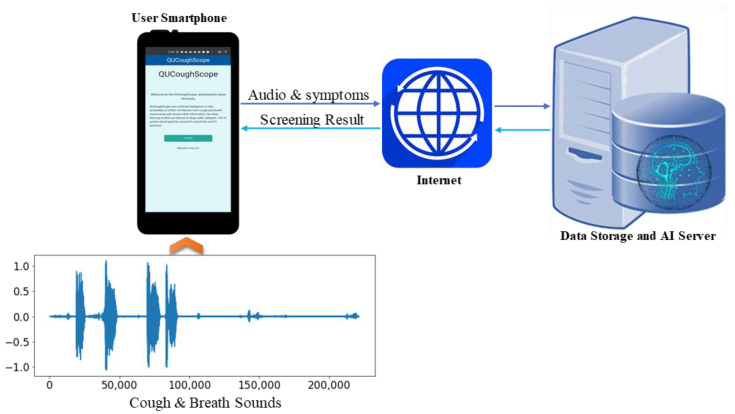
Illustration of a generic framework for the QUCoughScope application.

In the prototype system, the user fills in some demographic data, as well as a list of confirmed symptoms. Next, once the app collects cough and breath sounds from the user, these are transferred to the server using HTTPS protocol. The server performs signal processing and machine learning classification to determine whether the cough and breath sounds like those of COVID-19 patients or not (Figure 7). Our app then notifies the users about their status. The application displays the results and also stores them in a cloud database.

Our pipeline is divided into two parts: symptomatic (cough) and asymptomatic users (no symptoms). Once the spectrogram is generated, our AI-enabled server checks whether the user has a cough or not, based on which two separate pipelines are carried out. If the user has entered that he/she has a cough, the symptomatic pipeline is activated. It was observed that differentiating between COVID-19-positive and healthy users based on symptomatic and asymptomatic patients’ cough sounds plays a more important role than breath sounds. 

## 4. Conclusions

This work presents a novel stacking approach with deep CNN models for the automatic detection of COVID-19 using cough and breath sound spectrogram images for symptomatic and asymptomatic patients. As can be seen in comparison Table 6, the proposed innovative stacking approach has provided the best performance compared to similar studies. 

The performance of eight different CNN models was evaluated for the classification of different studies: binary classification of healthy and COVID-19 using cough and breath sound spectrogram images for symptomatic and asymptomatic patients. The study also evaluated the performance of the stacking CNN model in which the top three models were used as a base learner, and predictions of those models were used to train a logistic regression-based meta learner classifier for the final decision. The stacking CNN model outperformed other networks and the best classification accuracy, sensitivity, and specificity for binary classification using cough sound spectrogram images with symptomatic and asymptomatic data were found to be 96.5%, 96.42%, and 95.47% and 98.85%, 97.01%, and 99.6%, respectively. The best classification accuracy, sensitivity, and specificity for binary classification with symptomatic and asymptomatic breath sound data were found to be 91.03%, 88.9%, 91.5%, 80.01%, 72.04%, and 82.67% respectively. Thus, it is clear that cough sounds spectrogram images are more reliable in detecting COVID-19 patients than breath sound spectrograms. Moreover, the network has shown the best performance in detecting the asymptomatic patients, who are unknowing super-spreaders. The proposed web application can also help in crowdsourcing more data and further increasing the robustness of the solution. Therefore, automatic COVID-19 detection using cough sound spectrogram images can play a crucial role in computer-aided diagnosis as a fast diagnostic tool, which can detect a significant number of people in the early stages and can reduce healthcare costs and burden significantly. 

The limitations of this work include (i) a less diverse dataset in terms of ethnicity, as the datasets are from the UK and Qatar, (ii) less intuitiveness of the application in terms of not being able to distinguish between cough or breath sounds from other sounds, even though we have an option for the user to confirm the recorded sound, (iii) the dataset has limited RT-PCR verified labelled data.

These limitations can be minimized in future work, as the application is being proposed to many doctors and government organizations (nationally and internationally) so that the network can be trained with a more diverse dataset to improve itself. Doctors and government organizations can help in providing RT-PCR labelled datasets, as this convenient solution can be a much better replacement of low sensitivity rapid antigen test kits, which are widely used for quicker results. The authors are working to train an anomaly detection model to ensure that the user can only submit cough and breathing sounds while other sounds will not be accepted. This will improve the robustness of the proposed system.

## Figures and Tables

**Table 1:** Details of the total Dataset.

Experiments	Healthy		COVID-19	
	Cambridge	QU	Cambridge	QU
Symptomatic (Cough/Breath)	264	32	54	18
Asymptomatic (Cough/Breath)	318	213	87	78
Total	582	245	141	96

**Table 2:** Experimental pipelines for this study.

Pipelines	COVID-19	Healthy
Pipeline I (Symptomatic)	a. Cough b. Breath	a. Cough b. Breath
Pipeline II (Asymptomatic)	a. Cough b. Breath	a. Cough b. Breath

**Table 3:** Number of mages per class and per fold used for different pipelines.

Categories	Classes	Total Samples	Training Samples	Validation Samples	Test Samples
Symptomatic **(Cough/Breath)**	Healthy	296	213 × 10 = 2130	24	59
	COVID-19	72	52 × 38 = 1976	6	14
Asymptomatic **(Cough/Breath)**	Healthy	531	383 × 5 = 1915	42	106
	COVID-19	165	119 × 17 = 2023	13	33

**Table 4:** Details of training parameters for classification.

	Training Parameters for Classification					
	Batch Size	Learning Rate	Number of Epochs	Epoch Patience	Stopping Criteria	Optimizer
Parameters	32	0.001	30	15	15	ADAM

**Table 5:** Comparison of different CNN performances for binary classification for symptomatic and asymptomatic patients’ (A) cough and (B) breath sounds.

**(A)**							
**Scheme**	**Network**	**Overall**	**Weighted 95% CI**				**Inference Time (Sec)**
		**Accuracy**	**Precision**	**Sensitivity**	***F*1-Score**	**Specificity**	
Symptomatic	Resnet18	93.20 ± 2.57	93.65 ± 2.49	93.21 ± 2.57	93.35 ± 2.55	89.94 ± 3.07	0.0024
	Resnet50	95.38 ± 2.14	95.41 ± 2.14	95.38 ± 2.14	95.39 ± 2.14	90.47 ± 3.00	0.0061
	Resnet101	94.29 ± 2.37	95.41 ± 2.14	94.29 ± 2.37	94.53 ± 2.32	97.56 ± 1.58	0.0108
	Inception_v3	90.76 ± 2.96	91.53 ± 2.84	90.76 ± 2.96	91.02 ± 2.92	86.19 ± 3.52	0.0238
	DenseNet201	93.25 ± 2.56	93.78 ± 2.47	93.21 ± 2.57	93.39 ± 2.54	90.99 ± 2.93	0.0258
	Mobilenetv2	90.49 ± 3.00	90.78 ± 2.96	90.49 ± 3.00	90.61 ± 2.98	81.92 ± 3.93	0.0055
	EfficientNet_B0	90.20 ± 2.89	90.15 ± 2.90	91.30 ± 2.88	91.20 ± 2.89	78.97 ± 4.16	0.0106
	EfficientNet_B7	91.30 ± 2.88	91.40 ± 2.86	91.31 ± 2.88	91.35 ± 2.87	82.12 ± 3.92	0.0428
	**Stacking CNN model**	**96.50 ± 1.88**	**96.30 ± 1.93**	**96.42 ± 1.90**	**96.32 ± 1.92**	**95.47 ± 2.12**	**0.0389**
Asymptomatic	Resnet18	96.70 ± 1.33	96.68 ± 1.33	96.69 ± 1.33	96.66 ± 1.33	92.29 ± 1.98	0.0027
	Resnet50	94.97 ± 1.62	95.12 ± 1.60	94.98 ± 1.62	94.80 ± 1.65	85.07 ± 2.65	0.0058
	Resnet101	96.84 ± 1.30	96.84 ± 1.30	96.84 ± 1.30	96.84 ± 1.30	94.42 ± 1.71	0.0121
	Inception_v3	96.26 ± 1.41	96.30 ± 1.40	96.27 ± 1.41	96.19 ± 1.42	89.65 ± 2.26	0.0235
	DenseNet201	98.28 ± 0.97	98.27 ± 0.97	96.28 ± 1.41	97.11 ± 1.24	99.20 ± 0.66	0.0260
	Mobilenetv2	98.50 ± 0.90	98.30 ± 0.96	96.45 ± 1.37	97.25 ± 1.21	99.20 ± 0.66	0.0052
	EfficientNet_B0	93.82 ± 1.79	93.74 ± 1.80	93.82 ± 1.79	93.72 ± 1.80	85.96 ± 2.58	0.0118
	EfficientNet_B7	95.40 ± 1.56	95.40 ± 1.56	95.40 ± 1.56	95.31 ± 1.57	88.13 ± 2.40	0.046
	**Stacking CNN model**	**98.85 ± 0.79**	**97.76 ± 1.10**	**97.01 ± 1.27**	**97.41 ± 1.18**	**99.6 ± 0.47**	**0.0411**
**(B)**							
**Scheme**	**Network**	**Overall**	**Weighted 95% CI**				**Inference Time (sec)**
		**Accuracy**	**Precision**	**Sensitivity**	***F*1-Score**	**Specificity**	
Symptomatic	Resnet18	81.49 ± 3.97	70.27 ± 4.67	82.27 ± 3.90	75.80 ± 4.38	81.49 ± 3.97	0.0027
	Resnet50	80.66 ± 4.04	70.83 ± 4.64	81.83 ± 3.94	75.93 ± 4.37	80.67 ± 4.03	0.0060
	Resnet101	84.53 ± 3.69	73.01 ± 4.54	84.01 ± 3.74	78.12 ± 4.22	84.53 ± 3.69	0.0098
	Inception_v3	81.49 ± 3.97	71.05 ± 4.63	82.05 ± 3.92	76.15 ± 4.35	81.49 ± 3.97	0.0254
	DenseNet201	83.98 ± 3.75	72.43 ± 4.57	83.43 ± 3.8	77.54 ± 4.26	83.98 ± 3.75	0.026
	Mobilenetv2	87.57 ± 3.37	69.50 ± 4.7	87.50 ± 3.38	77.47 ± 4.27	87.57 ± 3.37	0.0048
	EfficientNet_B0	90.33 ± 3.02	70.28 ± 4.67	90.28 ± 3.03	79.03 ± 4.16	90.33 ± 3.02	0.0104
	EfficientNet_B7	81.77 ± 3.94	70.99 ± 4.64	81.99 ± 3.93	76.09 ± 4.36	81.77 ± 3.94	0.0434
	**Stacking CNN model**	**91.03 ± 2.92**	**71.91 ± 4.59**	**88.9 ± 3.21**	**79.62 ± 4.12**	**91.5 ± 2.85**	**0.0265**
Asymptomatic	Resnet18	66.75 ± 3.50	53.95 ± 3.7	66.66 ± 3.50	59.64 ± 3.64	78.54 ± 3.05	0.0025
	Resnet50	66.67 ± 3.50	55.45 ± 3.69	66.67 ± 3.50	60.54 ± 3.63	75.27 ± 3.21	0.0047
	Resnet101	69.72 ± 3.41	56.45 ± 3.68	69.71 ± 3.41	62.38 ± 3.60	73.52 ± 3.28	0.0118
	Inception_v3	67.10 ± 3.49	57.10 ± 3.68	68.26 ± 3.46	62.18 ± 3.60	81.25 ± 2.90	0.0243
	DenseNet201	67.97 ± 3.47	55.91 ± 3.69	67.97 ± 3.47	61.35 ± 3.62	79.88 ± 2.98	0.0271
	MobileNetv2	68.40 ± 3.45	53.22 ± 3.71	67.10 ± 3.49	59.36 ± 3.65	78.54 ± 3.05	0.0048
	EfficientNet_B0	68.30± 3.46	57.45 ± 3.67	68.62 ± 3.45	62.54 ± 3.60	76.50 ± 3.15	0.0128
	EfficientNet_B7	75.60 ± 3.19	54.20 ± 3.70	72.59 ± 3.31	62.06 ± 3.61	80.20 ± 2.96	0.0511
	**Stacking CNN model**	**80.01 ± 2.97**	**56.02 ± 3.69**	**72.04 ± 3.33**	**63.3 ± 3.58**	**82.67 ± 2.81**	**0.0687**

**Table 6:** Comparison of the proposed work with similar studies.

Papers	Dataset	Phenomenon	Reported Method	Performance
N. Sharma (2020) [48]	Healthy and COVID-19-positive: 941	Cough, Breathing, Vowel, and Counting (1–20)	Random forest classifier using spectral contrast, MFCC, spectral roll-off, spectral centroid, mean square energy, polynomial fit, zero-crossing rate, spectral bandwidth, and spectral flatness.	Accuracy: 76.74%
C. Brown et al. (2021) [55]	COVID-19-positive: 141, Non-COVID: 298, COVID-19-positive with Cough: 54, Non-COVID-19 with Cough: 32, Non-COVID-19 asthma: 20	Cough and Breathing	CNN-based approach using spectrogram, spectral centroid, MFCC.	Accuracy: 80%
V. Espotovic (2021) [71]	COVID-19-Positive: 84, COVID-19-Negative: 419	Cough and Breathing	Ensemble-boosted approach using spectrogram and wavelet.	Accuracy: 88.52%
R.Islam (2022) [72]	COVID-19-Positve: 50, Healthy: 50	Cough	CNN-based approach using zero-crossing rate, energy, energy entropy, spectral centroid, spectral entropy, spectral flux, spectral roll-offs, MFCC.	Accuracy: 88.52%
Proposed Study	COVID-19-Positve: 237, Healthy: 827	Cough and Breathing	Stacking-based CNN based approach using spectograms	For symptomatic, accuracy: 96.5% and for asymptomatic, accuracy: 98.85%

## Data Availability

The dataset collected from QUCoughScope through crowdsourcing is available in [59].

## References

[1] COVID-19 CORONAVIRUS PANDEMIC. https://www.worldometers.info/coronavirus/.

[2] Pormohammad A., Ghorbani S., Khatami A., Farzi R., Baradaran B., Turner D.L., Turner R.J., Bahr N.C., Idrovo J.P. (2020). Comparison of confirmed COVID-19 with SARS and MERS cases-Clinical characteristics, laboratory findings, radiographic signs and outcomes: A systematic review and meta-analysis. Rev. Med. Virol..

[3] Felsenstein S., Hedrich C.M. (2020). COVID-19 in children and young people. Lancet Rheumatol..

[4] Sattar N., Ho F.K., Gill J.M., Ghouri N., Gray S.R., Celis-Morales C.A., Katikireddi S.V., Berry C., Pell J.P., McMurray J.J. (2020). BMI and future risk for COVID-19 infection and death across sex, age and ethnicity: Preliminary findings from UK biobank. Diabetes Metab. Syndr. Clin. Res. Rev..

[5] Wise J. (2021). COVID-19: UK cases of variant from India rise by 160% in a week. BMJ Br. Med. J..

[6] Jain V.K., Iyengar K., Vaish A., Vaishya R. (2020). Differential mortality in COVID-19 patients from India and western countries. Diabetes Metab. Syndr. Clin. Res. Rev..

[7] Scohy A., Anantharajah A., Bodéus M., Kabamba-Mukadi B., Verroken A., Rodriguez-Villalobos H. (2020). Low performance of rapid antigen detection test as frontline testing for COVID-19 diagnosis. J. Clin. Virol..

[8] Khandker S.S., Nik Hashim N.H.H., Deris Z.Z., Shueb R.H., Islam M.A. (2021). Diagnostic accuracy of rapid antigen test kits for detecting SARS-CoV-2: A systematic review and meta-analysis of 17,171 suspected COVID-19 patients. J. Clin. Med..

[9] Albert E., Torres I., Bueno F., Huntley D., Molla E., Fernández-Fuentes M.Á., Martínez M., Poujois S., Forqué L., Valdivia A. (2021). Field evaluation of a rapid antigen test (Panbio™ COVID-19 Ag Rapid Test Device) for COVID-19 diagnosis in primary healthcare centres. Clin. Microbiol. Infect..

[10] Chowdhury M.E., Khandakar A., Qiblawey Y., Reaz M.B.I., Islam M.T., Touati F. (2020). Machine learning in wearable biomedical systems. Sports Science and Human Health-Different Approaches.

[11] Chowdhury M.E., Rahman T., Khandakar A., Mazhar R., Kadir M.A., Mahbub Z.B., Islam K.R., Khan M.S., Iqbal A., Al Emadi N. (2020). Can AI help in screening viral and COVID-19 pneumonia?. IEEE Access.

[12] Rahman T., Khandakar A., Qiblawey Y., Tahir A., Kiranyaz S., Kashem S.B.A., Islam M.T., Al Maadeed S., Zughaier S.M., Khan M.S. (2021). Exploring the effect of image enhancement techniques on COVID-19 detection using chest X-ray images. Comput. Biol. Med..

[13] Tahir A., Qiblawey Y., Khandakar A., Rahman T., Khurshid U., Musharavati F., Islam M., Kiranyaz S., Chowdhury M. (2022). Deep Learning for Reliable Classification of COVID-19, MERS, and SARS from Chest X-Ray Images. Cogn. Comput..

[14] Tahir A.M., Chowdhury M.E., Khandakar A., Rahman T., Qiblawey Y., Khurshid U., Kiranyaz S., Ibtehaz N., Rahman M.S., Al-Madeed S. (2021). COVID-19 Infection Localization and Severity Grading from Chest X-ray Images. arXiv.

[15] Qiblawey Y., Tahir A., Chowdhury M.E., Khandakar A., Kiranyaz S., Rahman T., Ibtehaz N., Mahmud S., Maadeed S.A., Musharavati F. (2021). Detection and severity classification of COVID-19 in CT images using deep learning. Diagnostics.

[16] Zhao J., Zhang Y., He X., Xie P. (2020). COVID-ct-dataset: A ct scan dataset about COVID-19. arXiv.

[17] He X., Yang X., Zhang S., Zhao J., Zhang Y., Xing E., Xie P. (2020). Sample-efficient deep learning for COVID-19 diagnosis based on CT scans. medrxiv.

[18] Rahman T., Akinbi A., Chowdhury M.E., Rashid T.A., Şengür A., Khandakar A., Islam K.R., Ismael A.M. (2022). COV-ECGNET: COVID-19 detection using ECG trace images with deep convolutional neural network. Health Inf. Sci. Syst..

[19] Hasoon N., Fadel A.H., Hameed R.S., Mostafa S.A., Khalaf B.A., Mohammed M.A., Nedoma J. (2020). COVID-19 anomaly detection and classification method based on supervised machine learning of chest X-ray images. Results Phys..

[20] Alyasseri Z.A.A., Al-Betar M.A., Doush I.A., Awadallah M.A., Abasi A.K., Makhadmeh S.N., Alomari O.A., Abdulkareem K.H., Adam A., Damasevicius R. (2021). Review on COVID-19 diagnosis models based on machine learning and deep learning approaches. Expert Syst..

[21] Al-Waisy A., Mohammed M.A., Al-Fahdawi S., Maashi M., Garcia-Zapirain B., Abdulkareem K.H., Mostafa S.A., Le D.N. (2021). COVID-DeepNet: Hybrid multimodal deep learning system for improving COVID-19 pneumonia detection in chest X-ray images. Comput. Mater. Contin..

[22] Abdulkareem K.H., Mohammed M.A., Salim A., Arif M., Geman O., Gupta D., Khanna A. (2021). Realizing an effective COVID-19 diagnosis system based on machine learning and IOT in smart hospital environment. IEEE Internet Things J..

[23] Obaid O.I., Mohammed M.A., Mostafa S.A. (2020). Long Short-Term Memory Approach for Coronavirus Disease Predicti. J. Inf. Technol. Manag..

[24] Khan K.N., Khan F.A., Abid A., Olmez T., Dokur Z., Khandakar A., Chowdhury M.E., Khan M.S. (2020). Deep Learning Based Classification of Unsegmented Phonocardiogram Spectrograms Leveraging Transfer Learning. arXiv.

[25] Chowdhury M.E., Khandakar A., Alzoubi K., Mansoor S., Tahir A.M., Reaz M.B.I., Al-Emadi N. (2019). Real-time smart-digital stethoscope system for heart diseases monitoring. Sensors.

[26] Mu W., Yin B., Huang X., Xu J., Du Z. (2021). Environmental sound classification using temporal-frequency attention based convolutional neural network. Sci. Rep..

[27] Connolly J.H., Edmonds E.A., Guzy J., Johnson S., Woodcock A. (1986). Automatic speech recognition based on spectrogram reading. Int. J. Man-Mach. Stud..

[28] Arias-Vergara T., Klumpp P., Vasquez-Correa J.C., Noeth E., Orozco-Arroyave J.R., Schuster M. (2021). Multi-channel spectrograms for speech processing applications using deep learning methods. Pattern Anal. Appl..

[29] Badshah A.M., Ahmad J., Rahim N., Baik S.W. Speech emotion recognition from spectrograms with deep convolutional neural network. Proceedings of the 2017 International Conference on Platform Technology and Service (PlatCon).

[30] Deshpande G., Schuller B. (2020). An overview on audio, signal, speech, & language processing for COVID-19. arXiv.

[31] Nasreddine Belkacem A., Ouhbi S., Lakas A., Benkhelifa E., Chen C. (2020). End-to-End AI-Based Point-of-Care Diagnosis System for Classifying Respiratory Illnesses and Early Detection of COVID-19. arXiv.

[32] Schuller B.W., Schuller D.M., Qian K., Liu J., Zheng H., Li X. (2020). COVID-19 and computer audition: An overview on what speech & sound analysis could contribute in the SARS-CoV-2 corona crisis. arXiv.

[33] Pramono R.X.A., Bowyer S., Rodriguez-Villegas E. (2017). Automatic adventitious respiratory sound analysis: A systematic review. PLoS ONE.

[34] Li S.-H., Lin B.-S., Tsai C.-H., Yang C.-T., Lin B.-S. (2017). Design of wearable breathing sound monitoring system for real-time wheeze detection. Sensors.

[35] Oletic D., Bilas V. (2016). Energy-efficient respiratory sounds sensing for personal mobile asthma monitoring. IEEE Sens. J..

[36] Brabenec L., Mekyska J., Galaz Z., Rektorova I. (2017). Speech disorders in Parkinson’s disease: Early diagnostics and effects of medication and brain stimulation. J. Neural Transm..

[37] Erdogdu Sakar B., Serbes G., Sakar C.O. (2017). Analyzing the effectiveness of vocal features in early telediagnosis of Parkinson’s disease. PLoS ONE.

[38] Maor E., Sara J.D., Orbelo D.M., Lerman L.O., Levanon Y., Lerman A. (2018). Voice signal characteristics are independently associated with coronary artery disease. Mayo Clin. Proc..

[39] Banerjee D., Islam K., Xue K., Mei G., Xiao L., Zhang G., Xu R., Lei C., Ji S., Li J. (2019). A deep transfer learning approach for improved post-traumatic stress disorder diagnosis. Knowl. Inf. Syst..

[40] Faurholt-Jepsen M., Busk J., Frost M., Vinberg M., Christensen E.M., Winther O., Bardram J.E., Kessing L.V. (2016). Voice analysis as an objective state marker in bipolar disorder. Transl. Psychiatry.

[41] hui Huang Y., jun Meng S., Zhang Y., sheng Wu S., Zhang Y., wei Zhang Y., xiang Ye Y., feng Wei Q., gui Zhao N., ping Jiang J. (2020). The respiratory sound features of COVID-19 patients fill gaps between clinical data and screening methods. medRxiv.

[42] Imran A., Posokhova I., Qureshi H.N., Masood U., Riaz M.S., Ali K., John C.N., Hussain M.I., Nabeel M. (2020). AI4COVID-19: AI enabled preliminary diagnosis for COVID-19 from cough samples via an app. Inform. Med. Unlocked.

[43] Cohen-McFarlane M., Goubran R., Knoefel F. (2020). Novel coronavirus cough database: NoCoCoDa. IEEE Access.

[44] Grant D., McLane I., West J. Rapid and scalable COVID-19 screening using speech, breath, and cough recordings. Proceedings of the 2021 IEEE EMBS International Conference on Biomedical and Health Informatics (BHI).

[45] Mouawad P., Dubnov T., Dubnov S. (2021). Robust Detection of COVID-19 in Cough Sounds. SN Comput. Sci..

[46] Erdoğan Y.E., Narin A. (2021). COVID-19 detection with traditional and deep features on cough acoustic signals. Comput. Biol. Med..

[47] Pahar M., Klopper M., Warren R., Niesler T. (2021). COVID-19 Cough Classification using Machine Learning and Global Smartphone Recordings. Comput. Biol. Med..

[48] Sharma N., Krishnan P., Kumar R., Ramoji S., Chetupalli S.R., Ghosh P.K., Ganapathy S. (2020). Coswara--A Database of Breathing, Cough, and Voice Sounds for COVID-19 Diagnosis. arXiv.

[49] COVID-19 Screening by Cough Sound Analysis. https://coughtest.online.

[50] Pal A., Sankarasubbu M. Pay attention to the cough: Early diagnosis of COVID-19 using interpretable symptoms embeddings with cough sound signal processing. Proceedings of the 36th Annual ACM Symposium on Applied Computing.

[51] Bagad P., Dalmia A., Doshi J., Nagrani A., Bhamare P., Mahale A., Rane S., Agarwal N., Panicker R. (2020). Cough against COVID: Evidence of COVID-19 signature in cough sounds. arXiv.

[52] Laguarta J., Hueto F., Subirana B. (2020). COVID-19 artificial intelligence diagnosis using only cough recordings. IEEE Open J. Eng. Med. Biol..

[53] Belkacem A.N., Ouhbi S., Lakas A., Benkhelifa E., Chen C. (2021). End-to-End AI-Based Point-of-Care Diagnosis System for Classifying Respiratory Illnesses and Early Detection of COVID-19: A Theoretical Framework. Front. Med..

[54] Rahman M.A., Hossain M.S., Alrajeh N.A., Gupta B. (2021). A multimodal, multimedia point-of-care deep learning framework for COVID-19 diagnosis. ACM Trans. Multimid. Comput. Commun. Appl..

[55] Brown C., Chauhan J., Grammenos A., Han J., Hasthanasombat A., Spathis D., Xia T., Cicuta P., Mascolo C. Exploring automatic diagnosis of COVID-19 from crowdsourced respiratory sound data. Proceedings of the 26th ACM SIGKDD International Conference on Knowledge Discovery & Data Mining.

[56] Coppock H., Gaskell A., Tzirakis P., Baird A., Jones L., Schuller B. (2021). End-to-end convolutional neural network enables COVID-19 detection from breath and cough audio: A pilot study. BMJ Innov..

[57] Kumar L.K., Alphonse P. (2021). Automatic Diagnosis of COVID-19 Disease using Deep Convolutional Neural Network with Multi-Feature Channel from Respiratory Sound Data: Cough, Voice, and Breath. Alex. Eng. J..

[58] Orlandic L., Teijeiro T., Atienza D. (2021). The COUGHVID crowdsourcing dataset, a corpus for the study of large-scale cough analysis algorithms. Sci. Data.

[59] https://www.kaggle.com/tawsifurrahman/qucoughscope-covid19-cough-dataset.

[60] Rahman T., Khandakar A., Kadir M.A., Islam K.R., Islam K.F., Mazhar R., Hamid T., Islam M.T., Kashem S., Mahbub Z.B. (2020). Reliable tuberculosis detection using chest X-ray with deep learning, segmentation and visualization. IEEE Access.

[61] Tahir A., Qiblawey Y., Khandakar A., Rahman T., Khurshid U., Musharavati F., Islam M., Kiranyaz S., Chowdhury M.E. (2020). Coronavirus: Comparing COVID-19, SARS and MERS in the eyes of AI. arXiv.

[62] Rahman T., Chowdhury M.E., Khandakar A., Islam K.R., Islam K.F., Mahbub Z.B., Kadir M.A., Kashem S. (2020). Transfer learning with deep convolutional neural network (CNN) for pneumonia detection using chest X-ray. Appl. Sci..

[63] Chowdhury M.E., Rahman T., Khandakar A., Al-Madeed S., Zughaier S.M., Doi S.A., Hassen H., Islam M.T. (2021). An early warning tool for predicting mortality risk of COVID-19 patients using machine learning. Cogn. Comput..

[64] Overfitting in Machine Learning: What It Is and How to Prevent It. https://elitedatascience.com/overfitting-in-machine-learning.

[65] ResNet, AlexNet, VGGNet, Inception: Understanding Various Architectures of Convolutional Networks. https://cv-tricks.com/cnn/understand-resnet-alexnet-vgg-inception/.

[66] DenseNet: Better CNN Model than ResNet. http://www.programmersought.com/article/7780717554/.

[67] Sandler M., Howard A., Zhu M., Zhmoginov A., Chen L.-C. Mobilenetv2: Inverted residuals and linear bottlenecks. Proceedings of the IEEE Conference on Computer Vision and Pattern Recognition.

[68] Tan M., Le Q. Efficientnet: Rethinking model scaling for convolutional neural networks. Proceedings of the International Conference on Machine Learning.

[69] Khandakar A., Chowdhury M.E., Reaz M.B.I., Ali S.H.M., Hasan M.A., Kiranyaz S., Rahman T., Alfkey R., Bakar A.A.A., Malik R.A. (2021). A machine learning model for early detection of diabetic foot using thermogram images. Comput. Biol. Med..

[70] QUCoughScope. https://nibtehaz.github.io/qu-cough-scope/.

[71] Despotovic V., Ismael M., Cornil M., Mc Call R., Fagherazzi G. (2021). Detection of COVID-19 from voice, cough and breathing patterns: Dataset and preliminary results. Comput. Biol. Med..

[72] Islam R., Abdel-Raheem E., Tarique M. (2022). A study of using cough sounds and deep neural networks for the early detection of COVID-19. Biomed. Eng. Adv..

